# Patient-reported outcomes in breast cancer FDA drug labels and review documents

**DOI:** 10.1186/s41687-021-00308-y

**Published:** 2021-04-21

**Authors:** Kyungwan Hong, Kayleigh R. Majercak, Ester Villalonga-Olives, Eleanor M. Perfetto

**Affiliations:** 1 Department of Pharmaceutical Health Services Research, University of Maryland, School of Pharmacy, 220 Arch Street, 12th Floor, Room: 01-328, Baltimore, MD 21201 USA; 2 National Health Council, Washington DC, USA

## Abstract

**Background:**

Patient-reported outcomes (PROs) can provide valuable information about drug benefit-risk tradeoffs from the patient perspective and are particularly important to patients with breast cancer due to its symptoms and adverse events from breast cancer treatments. The United States Food and Drug Administration (U.S. FDA) has acknowledged PROs as important approval endpoints used in clinical trials of cancer drugs. However, previous studies found that PROs are rarely mentioned in cancer drug labels, a widely used and trusted source of information about drugs. Our objectives were to compare PRO data reported in FDA labeling versus FDA medical review documents for breast cancer drugs approved in the U.S. between 2000 and 2019 to identify possible causes for PRO-data labeling exclusions.

**Methods:**

We included new molecular entities (NMEs) and biologic license applications (BLAs) initially approved for breast cancer treatment by the FDA between 1/1/2000 and 12/31/2019. Product labeling and FDA medical review documents were collected from the FDA-Approved Drugs database (Drugs@FDA). From these resources, details on PRO measures used in trials, design of trials using PRO measures, PRO-endpoint status, analytical methods, and FDA reviewer comments regarding PRO measurement were extracted.

**Results:**

Of 633 FDA-approved drugs, 13 were indicated for breast cancer treatment; none of their prescribing information contained information about PROs. However, 11 of 13 (85%) included PRO measures and endpoint information in FDA medical review documents. PRO measures were used in 14 different clinical trials, and FDA reviewers’ comments regarding PRO measurement were related to lack of meaningfulness and clinical significance, lack of content validity, and inadequate analytical methods.

**Conclusions:**

Despite the importance of PROs to patients with breast cancer, PRO measures were only described in FDA medical review documents of breast cancer drugs, but not in drug product labeling. Therefore, it appears that PRO data are often collected in breast cancer trials, but have not been methodologically acceptable to FDA reviewers. Collaborative efforts between the FDA and industry are warranted to increase the number of breast cancer drug applications with appropriate use of PRO measures and endpoints.

## Introduction

Prescribing information and other labeling are documents prepared by pharmaceutical companies and approved by the United States Food and Drug Administration (U.S. FDA). Prescribing information delivers key safety and efficacy information about prescription drugs and biological products [1–3]. Among different efficacy endpoints used in clinical trials of cancer drugs, patient-reported outcomes (PROs), a type of clinical outcome assessment (COA), which measures the way a patient feels or functions directly from the patient without interpretation by other people, have been acknowledged by the FDA as an important approval endpoint [4, 5]. Therefore, a PRO measure is a useful tool for understanding the patient’s perspective on treatments and their benefits in ways that healthcare professionals are often unaware [6].

The FDA has put growing efforts into incorporating patient perspectives in drug development and evaluation. The FDA released the draft version of a PRO guidance for industry in 2006 and the final version in 2009 [7–9], which explains key elements and properties of PRO-instrument development. When the FDA Safety and Innovation Act (FDASIA) was signed in 2012 [10], the FDA established the patient-focused drug development (PFDD) initiative, which focuses on gathering patients’ perspectives on their conditions and treatments more systematically [9, 11, 12]. Also, the passage of the 21st Century Cures Act in 2016 required the FDA to issue new guidance documents on patient experience data and their role in drug development [9, 13]. Consequently, the FDA released the first of the series of four guidance documents on PFDD in June 2020, covering approaches for patient-experience data collection [14].

In breast cancer, patient perspectives and PROs are particularly important. The number of new cases of breast cancer in the U.S. reached approximately 270,000 in 2019, making breast cancer the most common cancer among women [15]. Also, in 2019 alone, nearly 40,000 women died from breast cancer in the U.S. [15]. Symptoms of breast cancer include, but are not limited to, breast ulceration, fatigue, and pain in different parts of the body (e.g., back, chest, axillary, etc.) [16]. Multiple studies identified that these symptoms cause significant physical and psychological burdens to breast cancer patients and their caregivers [17, 18].

Furthermore, according to the Voice of the Patient report on breast cancer, which summarizes information obtained through an FDA- or externally led meeting to understand patient perspectives for those living with breast cancer, adverse events from breast cancer treatments also significantly impact patients’ daily lives [19]. Adverse events of breast cancer treatments (e.g., nausea, vomiting, cognitive dysfunctions, and hair loss) are also known to decrease the breast cancer patients’ quality of life [20]. Therefore, when patients and healthcare professionals make treatment decisions, PROs can provide valuable information about trade-offs between benefits and adverse events of breast cancer treatments and reduce the burdens from breast cancer symptoms and adverse events of breast cancer treatments simultaneously.

However, previous studies report that PROs are rarely mentioned in labels of different cancer drugs including breast cancer drugs [21, 22]. For example, Gnanasakthy et al. investigated the labels of 40 drugs approved by the FDA Office of Hematology & Oncology Products between 2010 and 2014 and found only three had PRO claims in the labeling [21]. Similarly, Hao et al. examined labels and approval documents of 16 drugs commonly used for breast cancer treatment in 2016. They identified none had PRO claims in their labeling, whereas 11 had PRO data presented in their drug approval documents [22].

These studies suggest that PROs may be collected in drug development, but not included in labeling. Since labeling needs to deliver key safety and efficacy information about drugs concisely, labeling often lack details compared to journal publications and trial documents such as study protocols and clinical study reports. However, such a gap may have significant clinical implications because the labeling (i.e., a trustworthy source of information about drugs) should deliver the information required to achieve what is best for patients. It is unclear how often information about PROs is excluded in labeling and reasons for exclusion, which may not be solely due to the need for conciseness. Therefore, this study’s objectives are to compare the PRO-endpoint data reported in FDA approved labeling with that reported in FDA review documents for breast cancer drugs approved in the U.S. between 2000 and 2019 and identify potential reasons for exclusion of PRO data in labeling.

## Methods

This study included new molecular entities (NMEs) and biologic license applications (BLAs) initially approved for the treatment of any types of breast cancer by the FDA between January 1, 2000 and December 31, 2019. To identify these products, the “*Compilation of the Center for Drug Evaluation and Research (CDER) NME Drug and New Biologic Approvals*” from the FDA was used [23]. The document summarizes all the NMEs & BLAs approved by the FDA from 1985 to 2019. The study period was selected to have a sufficiently long timeline to observe how PROs were measured and used in breast cancer drug development. Furthermore, even before the release of the final PRO guidance in 2009 (and before the draft version of the guidance in 2006), multiple stakeholders including the FDA released recommendations on the concept and methodological considerations for PRO measures and PRO labeling claims [24, 25].

After identifying eligible products, documents were collected from Drugs@FDA, the FDA-Approved Drugs database, for each product: original labeling, the latest version of labeling (as of March 15, 2020), and FDA medical review documents (i.e., medical review and multi-discipline review). Since labeling can change over time, both original labeling and the latest version of labeling were examined to capture any difference due to revisions. The medical review documents contain the FDA’s evaluation of the trial documents (e.g., study protocols, clinical study reports, and etc.) submitted by manufacturers for market approval. Information includes but is not limited to objectives, design, and research methods for pivotal trials and data supporting safety/efficacy of the study medications. Therefore, the documents can provide information about clinical trials beyond labeling and journal publications [26, 27] and enable researchers to understand regulators’ perspectives through the comments and summaries.

From these documents, the following information about PRO measures and endpoints were searched and extracted: a) name of the PRO measure, b) design and characteristics of the clinical trial in which it was used, c) PRO-endpoint status (e.g., primary, secondary, or exploratory endpoint), d) analytical methods for PRO data analysis, and e) comments from FDA reviewers on deficiencies related to the PRO measure or endpoint (if applicable). Analytical methods for PRO data analysis and FDA reviewers’ comments were extracted to understand how the PRO measure was utilized and identify potential causes for exclusion from the labeling. These analytical methods include but are not limited to comparison of time to deterioration/progression and comparison of score changes on PRO measures from baseline.

After identifying PRO measures from labeling and medical review documents, each PRO measure was classified as symptom-related, function-related, or both. These categories were created based on concepts of interest commonly measured by different PRO measures [8, 28, 29]. Symptom-related PRO measures evaluate specific disease symptoms such as pain. Function-related PRO measures would focus on patients’ various functional status (e.g., physical and cognitive functions). If PRO measures incorporate the range of measures of symptoms as well as functioning in physical, mental, and social domains of health, we classified them as both. The classification was based on PRO measures’ psychometric characteristic studies and descriptions from the review documents. All data were extracted by two authors (KH and KM) independently, and any discrepancies were resolved through discussion. All data extraction and descriptive analyses of this study were done using Microsoft Excel 2016.

## Results

The FDA approved a total of 633 NMEs and BLAs between 2000 and 2019. Of the 633 drugs, 13 were initially approved for breast cancer treatment (Table 1). Among the 13, none of their original labels had any information about PRO measures or endpoints. Also, there were no differences between the original and the latest version of labels for the drugs reviewed, in terms of PRO measures and/or endpoints. However, review documents of 11 of the 13 breast cancer drugs (85%) included information about PRO measures and/or endpoints (Table 2).

**Table 1:** Food and Drug Administration breast cancer NMEs and BLAs approved 2000–2019 (*N* = 13)

Brand name	Generic name	NDA or BLA^**a**^	Market approval date	Approval designation	Indicated breast cancer types^**a**^
Faslodex	Fulvestrant	NDA	04/2002	Standard	HR+ metastatic breast cancer
Tykerb	Lapatinib	NDA	03/2007	Priority, Fast track	HER2+ advanced or metastatic breast cancer
Ixempra	Ixabepilone	NDA	10/2007	Priority	Locally advanced or metastatic breast cancer
Halaven	Eribulin mesylate	NDA	11/2010	Priority, Fast track	Metastatic breast cancer
Perjeta	Pertuzumab	BLA	06/2012	Priority	HER2+ metastatic breast cancer
Kadcyla	Ado-trastuzumab emtansine	BLA	02/2013	Priority, Fast track	HER2+ metastatic breast cancer
Ibrance	Palbociclib	NDA	02/2015	Priority, Accelerated, Breakthrough	ER+ and HER2- advanced breast cancer
Kisqali	Ribociclib	NDA	03/2017	Priority, Breakthrough	HR+ and HER2- advanced or metastatic breast cancer
Nerlynx	Neratinib	NDA	07/2017	Standard	HER2+ early-stage breast cancer
Verzenio	Abemaciclib	NDA	09/2017	Priority, Breakthrough, Fast track	HR+ and HER2- advanced or metastatic breast cancer
Talzenna	Talazoparib	NDA	10/2018	Priority	HER2-, germline BRCA mutated locally advanced or metastatic breast cancer
Piqray	Alpelisib	NDA	05/2019	Priority	HR+, HER2-, PIK3CA mutated, advanced or metastatic breast cancer
Enhertu	Fam-trastuzumab deruxtecan-nxki	BLA	12/2019	Priority, Accelerated, Breakthrough, Fast track	HER2+ unresectable or metastatic breast cancer

^a^Explanation of abbreviations: *NDA* New drug applications, *BLA* Biologic license application, *HR* Hormone receptor, *HER2* Human epidermal growth factor receptor 2, *ER* Estrogen receptor, *BRCA* Breast cancer susceptibility gene, *PIK3CA* Catalytic α-subunit of phosphatidylinositol-3-kinase

**Table 2:** Reporting of patient-reported outcome in prescribing information labeling versus medical review documents

	N (%)
**Original labeling**	0 / 13 (0%)
**Most recent labeling**	0 / 11^a^ (0%)
**Medical Review documents**	11 / 13 (85%)

^a^Original prescribing information labeling of two drugs (Piqray, Enhertu) were the latest versions

From the review documents of the 11 breast cancer drugs, nine different PRO measures were identified (Table 3). Of the nine PRO measures, six were classified as measuring both symptoms and functions (EORTC QLQ-BR23 [30], EORTC QLQ-C30 [31], EQ-5D [32], EQ-5D-5 L [33], FACT-B [34], FACT-B TOI [34, 35]), and three were classified as symptom-related (Modified brief pain inventory-short form (mBPI-sf) [36], Brief Pain Inventory short form (BPI-sf) [37, 38], FACT-B Breast Symptom Index (FBSI) [39]).

**Table 3:** Drugs with patient-reported outcome measure findings in Food and Drug Administration medical review documents

Brand name^**a**^	PRO measure^**b**^	Classification (Symptom, function, or both)	Clinical trials that used PRO measures	Trial design	PRO endpoint status	Analytical methods of PRO data^b^
Faslodex (Fulvestrant)	FACT-B TOI	Both	Trial #20	Phase 3, Randomized, Open label, Active-controlled trial	Secondary	Time to deterioration in QoL
Faslodex (Fulvestrant)	FACT-B TOI	Both	Trial #21	Phase 3, Randomized, Double-blind, Active-controlled trial	Secondary	Time to deterioration in QoL, Change in TOI
Tykerb (Lapatinib)	FACT-B, EQ-5D	Both	EGF 100151	Phase 3, Randomized, Open label, Active-Controlled trial	Secondary	Change in QoL
Tykerb (Lapatinib)	FACT-B	Both	EGF 20008	Phase 2, Open label, Uncontrolled, Two-cohort trial	Secondary	Change in QoL
Ixempra (Ixabepilone)	FBSI	Symptom	Study 046	Phase 3, Randomized, Open label, Active-controlled trial	Secondary	Changes in symptoms during therapy
Perjeta (Pertuzumab)	FACT-B TOI	Both	CLEOPATRA	Phase 3, Randomized, Double-blind, Placebo-controlled trial	Secondary	Time to symptom progression
Kadcyla (Ado-trastuzumab emtansine)	FACT-B TOI	Both	EMILIA	Phase 3, Randomized, Open label, Active-controlled trial	Secondary	Time to symptom progression
Ibrance (Palbociclib)	mBPI-sf	Symptom	PALOMA-1	Phase 1/2, Randomized, Open label, Active-controlled trial	Secondary	Changes in symptoms during therapy (pain)
Kisqali (Ribociclib)	EORTC QLQ-C30, EORTC QLQ-BR23, EQ-5D-5 L	Both	MONALEESA-2	Phase 3, Randomized, Double-blind, Placebo-controlled trial	Secondary	Time to deterioration in QoL, Change in QoL
Nerlynx (Neratinib)	EQ-5D, FACT-B	Both	Study 3004	Phase 3, Randomized, Double-blind, Placebo-controlled trial	Exploratory	Change in QoL
Verzenio (Abemaciclib)	mBPI-sf, EORTC QLQ-C30, EORTC QLQ-BR23, EQ-5D-5 L	Symptom (mBPI-sf), Both (rest)	MONARCH 2	Phase 3, Randomized, Double-blind, Placebo-controlled trial	Secondary	Time to symptom progression, Change in QoL and symptom
Verzenio (Abemaciclib)	mBPI-sf, EORTC QLQ-C30	Symptom (mBPI-sf), Both (rest)	MONARCH 1	Phase 2, Open label, Single arm trial	Secondary	Change in QoL and symptom
Talzenna (Talazoparib)	EORTC QLQ-C30, EORTC QLQ-BR23	Both	EMBRACA	Phase 3, Randomized, Open label, Active-controlled trial	Exploratory	Time to deterioration in QoL and symptom
Piqray (Alpelisib)	BPI-sf, EORTC QLQ-C30, EQ-5D-5 L	Symptom (BPI-sf), Both (rest)	SOLAR-1	Phase 3, Randomized, Double-blind, Placebo-controlled trial	Secondary	Time to deterioration in QoL, symptom, and functioning

^a^Review documents of Halaven and Enhertu did not include information about PRO measures and/or endpoints

^b^Explanation of abbreviations: *BPI-sf* Brief Pain Inventory short form, *EORTC QLQ-BR23* European Organization for Research and Treatment of Cancer, Breast Cancer-Specific Quality of Life Questionnaire, *EORTC QLQ-C30* European Organization for Research and Treatment of Cancer Quality of Life Questionnaire – Core Questionnaire, *EQ-5D* EuroQol 5 Dimensions, *EQ-5D-5 L* The 5-level EuroQol 5 Dimensions, *FACT-B TOI* Functional Assessment of Cancer Therapy-Breast Trial Outcome Index, *FBSI* Functional Assessment of Cancer Therapy-Breast Symptom Index, *mBPI-sf* Modified version of the Brief Pain Inventory short form, *QoL* Quality of Life, *TOI* Treatment Outcome Index

These PRO measures were used as an outcome measure in 14 different clinical trials. Of the 14 trials, 11 (79%) were phase 3, and 6 (43%) were double-blind trials. Only one (7%) trial was a single-arm trial. Among trials with comparators, seven (54%) were active-controlled, and five (38%) were placebo-controlled. One trial (8%) compared different doses of the same treatment.

None of the clinical trials used PRO measures as the primary endpoint. PRO measures were used as either a secondary endpoint (*N* = 12, 86%) or an exploratory endpoint (*N* = 2, 14%). Of the 14 trials, five (36%) analyzed their PRO data by comparing time to HRQoL deterioration or symptom progression, and six (43%) analyzed by comparing score changes from baseline. Three (21%) analyzed their PRO data using both methods. Four (29%) described strategies for handling the missing values in PRO measures. Two excluded the data from the analysis if there were missing values (Nerlynx: Study 3004, Talzenna: EMBRACA), and the other two used proration, unless 50% or more questions are unanswered (Faslodex: Trial #20 and Trial #21).

Comments from FDA reviewers were based on a variety of issues related to PRO measures and analytical methods of PRO data that are potential causes for exclusion of PRO information from the labeling (Table 4). FDA reviewers indicated that some PRO measures had quality issues such as lack of content validity or sensitivity to detect differences in symptoms between treatment arms. For example, FDA reviewers commented in the Perjeta medical review that, “It is questionable whether the FACT-B questionnaire has content validity.” In the Ibrance medical review, FDA reviewers also commented that “The mBPI-sf is not a comprehensive or sensitive PRO instrument” Moreover, reviewers commented on PRO data collection and analysis identifying issues such as missing type-I error adjustment or alpha allocation and having low statistical power. For example, FDA reviewers commented in Kisqali multi-disciplinary review that, “Patient-reported outcomes were not allocated alpha so any results should be considered exploratory.” Furthermore, reviewers suggested it was difficult to interpret and reach a meaningful conclusion from the provided PRO data in multiple review documents due to study design and characteristics of PRO measures. For example, the FDA reviewers commented, “No conclusions can be reached from the PRO data. The pre-specified design did not take into account the possibility of loss of respondents due to an inferior therapy” in the Ixempra medical review. Also, in Talzenna multi-disciplinary review, FDA reviewers commented, “PRO outcomes are likely to be biased [due to the open-label trial design].” In Verzenio multi-disciplinary review, FDA reviewers specifically recommended not to include PRO data in labeling due to the lack of prespecified objectives.

**Table 4:** Food and Drug Administration reviewer comments on deficiencies related to patient-reported outcome measures

Brand name (Generic name)^**a**^	PRO Measure	Approval year	Deficiencies related to PRO measures/findings commented by FDA reviewers^**b**^
Faslodex (Fulvestrant)	FACT-B TOI	2002	• Insufficient data collection • Difficult to reach a meaningful conclusion
Tykerb (Lapatinib)	FACT-B, EQ-5D	2007	• No comments
Ixempra (Ixabepilone)	FBSI	2007	• Difficult to reach a clinically meaningful conclusion • Poor compliance after the baseline evaluation
Perjeta (Pertuzumab)	FACT-B TOI	2012	• Questionable content validity • Questionable sensitivity to detect differences in symptoms between treatment arms due to long interval between questionnaire administration.
Kadcyla (Ado-trastuzumab emtansine)	FACT-B TOI	2013	• No comments
Ibrance (Palbociclib)	mBPI-sf	2015	• PRO measure is not comprehensive or sensitive • Open label study design complicates PRO interpretation.
Kisqali (Ribociclib)	EORTC QLQ-C30, EORTC QLQ-BR23, EQ-5D-5 L	2017	• PROs were not alpha allocated and considered exploratory.
Nerlynx (Neratinib)	EQ-5D, FACT-B	2017	• FACT-B’s combined assessments of disease symptoms and global health status complicates PRO interpretation. • Lack of clear guidance on different levels of severity • PRO data were no longer collected after Amendment 9.
Verzenio (Abemaciclib)	EORTC QLQ-C30, EORTC QLQ-BR23, mBPI-sf, EQ-5D-5 L	2017	• Lack of prespecified objectives • Treatment related symptom data do not add significantly to the existing adverse event data.
Talzenna (Talazoparib)	EORTC QLQ-C30, EORTC QLQ-BR23	2018	• PRO analysis was subjective and not validated in the patient population. • PRO outcomes are likely to be biased due to the open label study design (i.e., EMBRACA). • Missing type-1 error adjustment.
Piqray (Alpelisib)	BPI-sf, EQ-5D-5 L, EORTC QLQ-C30	2019	• PRO analysis was not controlled for multiple comparisons and considered exploratory. • Outcomes in PRO analysis was not clearly defined • Not adequately powered to support a non-inferiority conclusion.

^a^Review documents of Halaven and Enhertu did not include information about PRO measures and/or endpoints

^b^Abbreviated comments

## Discussion

For patients with breast cancer, chemotherapy can be challenging due to intolerable adverse events such as extreme fatigue, hair loss, and nausea [40]. Like breast cancer symptoms, these adverse events also prevent breast cancer patients from performing daily activities and cause significant burdens. Thus, PROs can provide additional insights on breast cancer treatment’s impact on changes in symptoms and physical/social functioning, which can be useful for both patients and healthcare professionals. In the current era of personalized cancer medicine, it is not surprising that PRO measures have been widely used in breast cancer populations [41, 42]. For example, Howell et al. reviewed the use of PRO measures in routine cancer clinical practice from 2003 to 2013 and suggested that PRO measures were used most often in breast cancer, among the five most prevalent cancers [43].

Interestingly, for the 13 breast cancer drugs we reviewed, approved from 2000 to 2019, none of their labelings included information on PROs. The reason for the lack of information on PROs in drug labels could be that pharmaceutical companies did not collect the PRO data, or they collected it, but the FDA did not accept the PRO data for inclusion the label. We found that for most of the 13 breast cancer drugs, PRO data were collected during drug development but were not included in the labels. Examination of FDA reviewers’ comments in review documents revealed the likely causes behind why the collected PRO data are not included in the drug labels.

Although the FDA reviewers commented on a variety of deficiencies related to PRO findings that do not appear to be consistent across measures and statistical methods, their comments focus on mainly three types of deficiencies: a) insufficient psychometric properties of PRO instruments (e.g., lack of content validity or sensitivity), b) inadequate analytical methods (e.g., missing error adjustment, insufficient statistical power), and c) lack of clarity on the provided PRO data (e.g., difficulty in interpretation or reaching a meaningful conclusion, lacking prespecified objectives). Sometimes, such deficiencies led to disagreements between industry and the FDA regarding PRO endpoints. For example, PROs measuring global quality of life were among the secondary endpoints of a Kisqali’s pivotal trial (i.e., MONALEESA-2), but FDA reviewers disagreed and considered the PROs as exploratory endpoints. These deficiencies align with potential reasons why the FDA may have rejected inclusion of PRO data for labeling claims suggested by previous studies that investigated review documents [44, 45]. For example, DeMuro et al. suggested that of the 116 NMEs and BLAs approved in 2006–2010, 52 collected PRO data in their pivotal trials, and 28 included information on PROs in their labels. The study found that reasons for the denial of PRO labeling claims include issues with being “fit for purpose” (38%); study design, data quality, and interpretation (27%); and inappropriate statistical analysis (11%) [45].

Our study’s findings suggest that breast cancer drugs approved as recently as 2019 still had these deficiencies in their PRO measure selection, data collection, and/or analyses. In particular, FDA reviewers commented on some PRO instruments’ lack of content validity (i.e., an extent to which an instrument’s content captures the intended concept of interest [8]). Evidence of content validity can be obtained from target population input in item generation (i.e., concept elicitation) and evaluation of patient understanding through cognitive interviews [46]. However, such deficiencies were pointed out even after the FDA released the final PRO guidance in 2009 [8] (The draft version of the PRO guidance was released in 2006 [7]). The draft and final guidances should have been guiding key elements and properties of PRO-instrument development and selection over the last 14 years.

Less commitment to PRO endpoints from the industry has been suggested to contribute to suboptimal compliance with PRO guidance [47]. For example, PROs can be used as either primary or secondary endpoints to support drugs’ efficacy and suggest treatment benefits for approval. Regardless of endpoint status, PROs need to be supported with scientifically sound methodology and satisfy rigorous evaluation criteria to be included in labeling [48, 49]. However, several studies suggest that the industry may invest fewer resources into non-primary endpoints in the early stages of product development [47, 50]. For example, Gnanasakthy et al. pointed out the likelihood of changes to the target product profile is higher during the early stages of product development and, therefore, may contribute to less commitment of resources into non-primary endpoints [47]. Considering PROs are often used as secondary endpoints, less commitment to PRO endpoints could have contributed to suboptimal compliance with the PRO guidance and resulting in a low number of PRO labeling claims. Also, Luckett et al. emphasized PRO selections and related details should be considered in the early phase of clinical trial designs as PROs should be supported with other methodological decisions such as research objectives, participant characteristics, and study interventions [51]. Therefore, the industry should provide enough supporting evidence along with PRO data and dedicate the same amount of attention and resources to PRO endpoints from the early stage of clinical trials study design and product development, even if they are non-primary endpoints.

Additionally, several studies state that inconsistencies within the FDA could contribute to such suboptimal compliance with the PRO guidances and lead to a low number of PRO labeling claims [52, 53]. For example, Fehnel et al. called attention to identified cases demonstrating that the FDA allowed certain drugs to have PRO labeling claims, although the PROs were not strictly compliant with the PRO guidance [53]. This inconsistency may be due to differences in perspectives between the FDA’s Division of Clinical Outcome Assessment (DCOA) and different FDA reviewing divisions. The FDA DCOA, formerly known as the Study Endpoints and Label Development (SEALD) team, assists reviewing divisions to make informed decisions regarding PRO labeling claims and tends to focus more on PRO measures’ developmental elements than reviewing divisions [53, 54]. The differing perspectives may have potentially led to inconsistent decisions regarding PRO labeling claims and confused the industry regarding being compliance with the 2009 PRO guidance [53].

With the passage of the 21st Century Cures Act in 2016, the FDA has begun to release required guidance documents related to patient engagement in drug development, including PROs [13, 14, 55]. These new guidances will replace the 2009 guidance when they are all released over the next few years. This is an essential milestone to clarify recommendations and promote more patient-focused drug development and patient-centered healthcare. In addition to the guidance documents, the FDA has provided suggestions on properly collecting PRO data in cancer trials and communicating the results more effectively through multiple journal publications. For example, Kluetz et al. suggest focusing on the core concepts of symptomatic adverse events, physical function, and disease symptoms [56, 57]. Fiero et al. examined the statistical analysis methods of PRO data used in clinical trials of lung cancer between 2008 and 2017 and recommended incorporating sensitivity analyses to address missing data and potential biases [58].

Additional research is needed to uncover the true, specific causes for there being no PRO-endpoint information included in breast cancer drug labeling over the last 20 years. There appears to be suboptimal compliance with the 2009 PRO guidance and additional recommendations. Nevertheless, it is unknown if that is because the guidance is not being followed by industry; if the guidance is unclear and, therefore, hard to follow; if the guidance is being interpreted differently by industry and/or across FDA divisions; or some combination of all of these explanations. If the FDA deemed the content validity or sensitivity of the PRO measures as lacking, providing additional justification and/or clarification to the industry may be a simple step to improve the compliance with the 2009 PRO guidance. Hence, a collaborative effort between the FDA and the industry is warranted to get to the heart of the issue(s). It would help to clarify the confusion around the use of PRO endpoints and promote PRO labeling claims. Efforts to improve the inclusion of PRO measure data in labels will be difficult to achieve without this information.

It is important to note that this study has several limitations. First, this study did not examine breast cancer drugs approved through supplemental new drug application (sNDAs) such as Tecentriq or Lynparza; thus, it does not provide a complete picture of PRO labeling claims in all breast cancer drugs during the study period. While their inclusion may have provided additional insights, Drugs@FDA is often inconsistent in providing regulatory information related to drugs approved through sNDA.

Second, this study only examined trials listed in medical review documents, which were mainly Phase 3 pivotal trials. Therefore, this study cannot make inferences about how PRO measures have been used in the entire breast cancer drug development process (e.g., Phase 1 or 2 trials, other study designs). Typically, Phase 1 and 2 trials do not include PROs or include them as part of PRO development and testing, as there is no intention to include the data in labeling.

Third, this study did not examine statistical methods used for PRO endpoints. In the medical review documents, details on the statistical methods used for PRO endpoints were often limited to the descriptions of PRO measures used and the endpoints. Also, they were less comprehensive than the description of primary endpoints, as all the identified PROs were used as either secondary or exploratory endpoints. Examination of trial documents such as statistical analysis plans, clinical study reports, or study protocols may provide further insight into the statistical methods used for comparing PRO data.

Notwithstanding the limitations, this study has several strengths. Compared to Hao et al., which previously examined labels and approval documents of 16 drugs commonly used for breast cancer treatment, this study examined all drugs initially approved for breast cancer treatment between 2000 and 2019. Therefore, this study provides a general overview of how PRO measures have been used and analyzed in breast cancer drug development specifically. This provides insight for future drug development endeavors in breast cancer. Not only was labeling investigated, but also the medical review documents. As a result, the review of these documents revealed some potential underlying causes that might have led to the exclusion of PRO information from the labeling.

## Conclusion

Despite the importance of PROs to patients with breast cancer, the labeling of breast cancer drugs lack information on PROs. PRO measures are often used in breast cancer drug development and described in medical review documents, but not included in labeling. This discrepancy appears to be because the PRO tools and/or analytical methods used for PRO measure data analysis have been considered inadequate by FDA reviewers. Research is needed investigating the specific causes for why PRO data have not been included in labeling and would likely be enhanced through collaborative efforts between the FDA and the industry. This is especially relevant as more PRO-related guidance documents (e.g., through patient-focused drug development) from the FDA are being released in the near future.

## Data Availability

The datasets used and/or analyzed are available from Drugs@FDA (https://www.accessdata.fda.gov/scripts/cder/daf/).
